# Prevalence of *Staphylococcus aureus* and Methicillin-Resistant *Staphylococcus aureus* in Retail Ready-to-Eat Foods in China

**DOI:** 10.3389/fmicb.2016.00816

**Published:** 2016-06-07

**Authors:** Xiaojuan Yang, Jumei Zhang, Shubo Yu, Qingping Wu, Weipeng Guo, Jiahui Huang, Shuzhen Cai

**Affiliations:** State Key Laboratory of Applied Microbiology Southern China, Guangdong Provincial Key Laboratory of Microbial Culture Collection and Application, Guangdong Open Laboratory of Applied Microbiology, Guangdong Institute of MicrobiologyGuangzhou, China

**Keywords:** *Staphylococcus aureus*, methicillin-resistant, ready-to-eat foods, prevalence, antibiotic resistance

## Abstract

*Staphylococcus aureus*, particularly methicillin-resistant *S*.aureus (MRSA), is a life-threatening pathogen in humans, and its presence in food is a public health concern. MRSA has been identified in foods in China, but little information is available regarding MRSA in ready-to-eat (RTE) foods. We aimed to investigate the prevalence of *S. aureus* and MRSA in Chinese retail RTE foods. All isolated *S. aureus* were tested for antimicrobial susceptibility, and MRSA isolates were further characterized by multilocus sequence typing (MLST) and staphylococcal cassette chromosome *mec* (SCC*mec*) typing. Of the 550 RTE foods collected from 2011 to 2014, 69 (12.5%) were positive for *S. aureus*. Contamination levels were mostly in the range of 0.3–10 most probable number (MPN)/g, with five samples exceeding 10 MPN/g. Of the 69 *S. aureus* isolates, seven were identified as MRSA by cefoxitin disc diffusion test. Six isolates were *mecA*-positive, while no *mecC*-positive isolates were identified. In total, 75.8% (47/62) of the methicillin-susceptible *S. aureus* isolates and all of the MRSA isolates were resistant to three or more antibiotics. Amongst the MRSA isolates, four were identified as community-acquired strains (ST59-MRSA-IVa (*n* = 2), ST338-MRSA-V, ST1-MRSA-V), while one was a livestock-associated strain (ST9, harboring an unreported SCC*mec* type 2C2). One novel sequence type was identified (ST3239), the SCC*mec* gene of which could not be typed. Overall, our findings showed that Chinese retail RTE foods are likely vehicles for transmission of multidrug-resistant *S. aureus* and MRSA lineages. This is a serious public health risk and highlights the need to implement good hygiene practices.

## Introduction

*Staphylococcus aureus* is an important cause of food poisoning worldwide. It is estimated that ~20–25% of foodborne bacterial outbreaks are caused by *S. aureus* in China (Wang et al., 2014). In addition, it is the leading cause of infection in both healthcare facilities and communities, causing illnesses ranging from mild skin and soft tissue infections to life threatening diseases such as septicemia, necrotizing fasciitis, endocarditis, and necrotizing pneumonia (Lowy, 1998; Chen and Huang, 2014; Rodríguez-Lázaro et al., 2015). Furthermore, the increasing antimicrobial resistance rates of this bacterium pose a serious threat to public health. Methicillin-resistant *S. aureus* (MRSA) strains exhibit resistance to all β-lactam antibiotics through acquisition of the mobile staphylococcal cassette chromosome *mec* (SCC*mec*), which carries the antibiotic-resistant gene *mecA*. Together, healthcare-acquired MRSA (HA-MRSA), community-acquired MRSA (CA-MRSA), and livestock-associated MRSA (LA-MRSA) strains constitute a major health concern.

Epidemiological studies have revealed differences between HA-MRSA, CA-MRSA, and LA-MRSA strains, including antimicrobial resistance profiles, SCC*mec* types, and clonal complexes (CCs) identified by multilocus sequence typing (MLST). HA-MRSA isolates typically carry relatively large SCC*mec* elements (types II or III), and are resistant to many classes of antimicrobials, including β-lactams (Yamamoto et al., 2013). CA-MRSA isolates usually harbor smaller SCC*mec* elements (types IV or V) and are only resistant to β-lactam antibiotics (Rodríguez-Lázaro et al., 2015). Interestingly, specific MRSA clones have spread across different geographical regions worldwide. The New York/Japan (ST5/SCC*mec*II), Brazilian/Hungarian (ST239/SCC*mec*III), EMRSA-15 (ST22/SCC*mec*IV), and EMRSA-16 (ST36/SCC*mec*II) clones are pandemic HA-MRSA lineages (Yamamoto et al., 2013), while the Taiwan (ST59/SCC*mec*IV or V), USA300 (ST8/SCC*mec*IV), European (ST80/SCC*mec*IV), and USA400 (ST1) clones are always associated with community-acquired infections (Yamamoto et al., 2013). In China, ST239/SCC*mec*III and ST5/SCC*mec*II are predominant HA-MRSA clones, while ST59/SCC*mec*IV or V is the most prevalent CA-MRSA clone (Chuang and Huang, 2013; Chen and Huang, 2014).

The first reports of MRSA infections in animals appeared in the 1970s and, on the basis of their putative source, these MRSA strains are referred to as LA-MRSA (Petinaki and Spiliopoulou, 2012). ST398 was the first detected and most widespread LA-MRSA sequence type (ST). The isolates belonging to this clonal lineage are not typeable by pulsed field gel electrophoresis (PFGE) using *Sma*I, and often exhibit co-resistance to many non-β-lactam antimicrobials, including those commonly used in animal production.

The emergence of LA-MRSA has led to concerns about its transmission via the food chain. In recent years, LA-MRSA has frequently been detected in food-producing animals (Wagenaar et al., 2009; Petinaki and Spiliopoulou, 2012; Visciano et al., 2014), meat (Rodríguez-Lázaro et al., 2015), milk and dairy products (Song et al., 2015), fish (Hammad et al., 2012), and ready-to-eat (RTE) food products (Hammad et al., 2012; Wang et al., 2014), causing a significant public health concern.

RTE foods that are consumed without further treatment, such as cooked meat and poultry, cold vegetable dishes, cold noodles, and fried rice, are popular in China. It has been reported that these food products are associated with the introduction of microbiological hazards, including *Listeria monocytogenes* (Chen M. T. et al., 2014), *Cronobacter* (Xu et al., 2015), and *Salmonella* (Yang et al., 2016). However, fewer data are available regarding the prevalence of *S. aureus* and MRSA in these foods. The aim of this study was to determine the prevalence, antibiotic resistance, and molecular characteristics of *S. aureus* and MRSA isolated from RTE food samples collected in China.

## Materials and methods

### Sample collection

**Figure 1:**
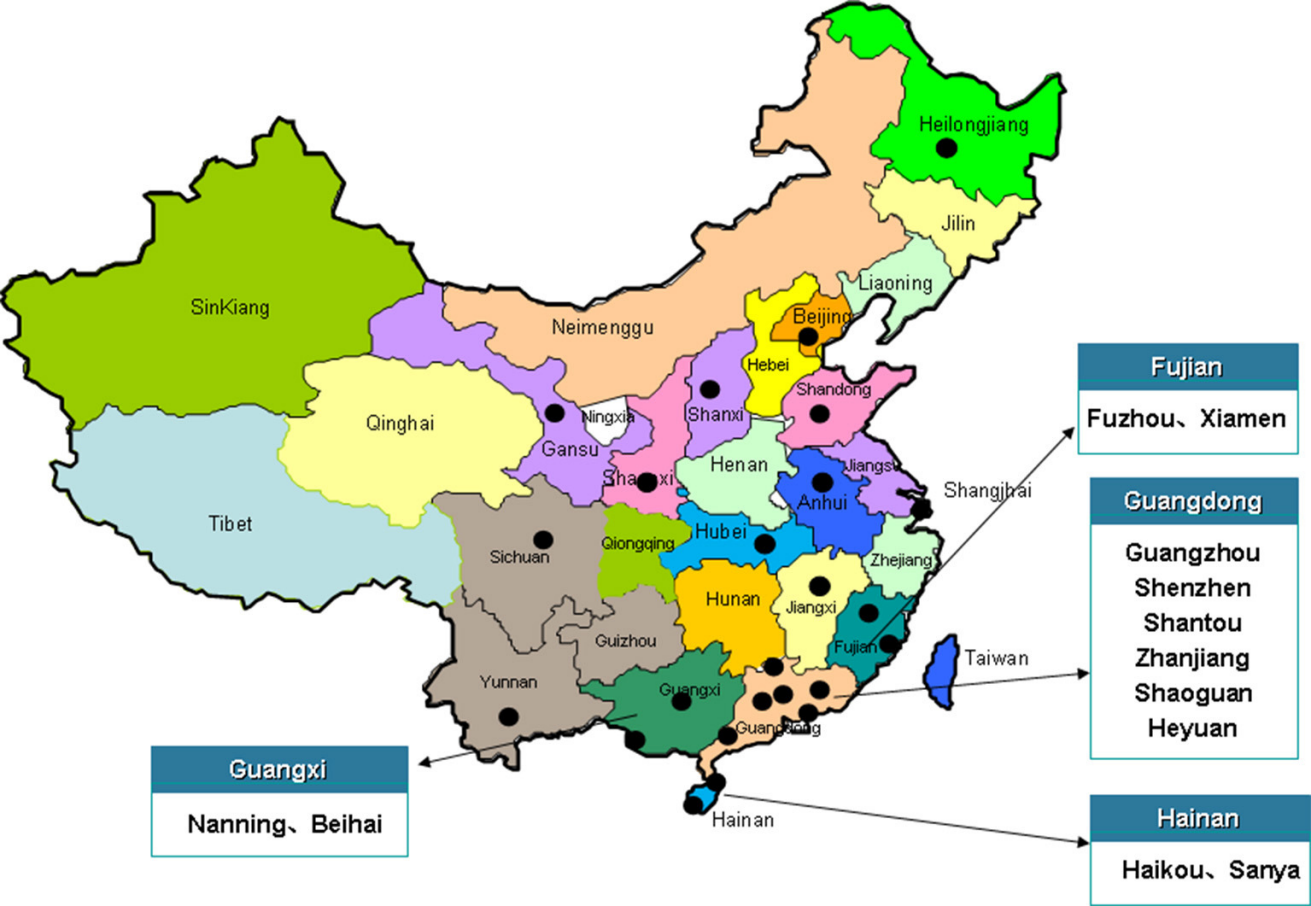
**Map of China showing the locations of the 24 cities where RTE samples were collected**.

From December 2011 to May 2014, a total of 550 samples were collected from RTE foods from retail markets. Samples included cooked pork (119), cooked chicken (153), cooked duck (127), cold vegetable dishes in sauce (53), cold noodles (52), and fried rice/sushi (46). The retail markets were located in 24 cities across most of the provincial capitals of China (Figure 1). Each sample was weighed, labeled, placed in a separate sterile bag, and then immediately transported to the laboratory in an ice box.

### Detection and enumeration of *S. aureus*

The prevalence and bacterial load of *S. aureus* in the samples was determined using the most probable number (MPN) method according to National Food Safety Standards of China document GB 4789.10-2010. A 25-g sample was randomly collected from each RTE food product and placed into a sterile glass flask containing 225 mL of saline solution (Huankai, Guangzhou, China). Following homogenization, trypticase soy broth (Huankai) supplemented with 10% NaCl was inoculated in triplicate with 1-ml aliquots of decimal dilutions of each sample. Broths were incubated at 37°C for 48 h. Loopfuls of the resulting cultures were streaked onto chromogenic *S. aureus* agar plates (Huankai), then incubated at 37°C for 24 h. Putative *S. aureus* isolates were tested for coagulase activity, and were further confirmed using API STAPH test strips (bioMerieux, Marcy-l′Etoile, France). The MPN value was determined on the basis of the number of positive tube(s) in each of the three sets using the MPN table.

### Antimicrobial susceptibility testing

All isolates were evaluated for antimicrobial resistance using the Kirby–Bauer disk diffusion method according to the (Clinical Laboratory Standards Institute previously National Committee on Clinical Laboratory Standards., 2014). Susceptibility to the following 16 antimicrobial agents was tested: ampicillin (AMP), cephalothin (KF), cefoxitin (FOX), penicillin G (P), chloramphenicol (C), tetracycline (TE), ciprofloxacin (CIP), amikacin (AK), gentamicin (CN), kanamycin (K), trimethoprim-sulfamethoxazole (SXT), erythromycin (E), clindamycin (DA), rifampicin (RD), linezolid (LZD), and quinupristin/dalfopristin (QD) (Oxoid, Basingstoke, UK). The isolates were also examined using a microdilution test according to the CLSI method for vancomycin minimum inhibitory concentrations (MICs) (Clinical Laboratory Standards Institute previously National Committee on Clinical Laboratory Standards., 2014).

### Detection of mecA and mecC

*mecA*, which has been shown to confer methicillin resistance on *S. aureus*, and *mecC*, a divergent *mecA* homolog (also called *mecALGA251*), were detected by PCR using primers described previously (Pérez-Roth et al., 2001; Stegger et al., 2012).

### MLST and SCCmec-typing of the mecA-positive MRSA isolates

The *mecA*-positive MRSA isolates were characterized by MLST analysis. MLST was carried out using previously reported primers specific for seven housekeeping genes (*arcC, aroE, glpF, gmk, pta, tpi*, and *yqiL*), and the sequence type (ST) was assigned according to the MLST database (http://www.mlst.net/).

The SCC*mec* type of *mecA*-positive strains was determined using a multiplex PCR method as previously described (Kondo et al., 2007). Strains with unanticipated fragments or completely lacking fragments were defined as non-typeable (NT).

## Results

### Prevalence and load of *S. aureus* in retail RTE food

Of the 550 retail RTE food samples, 69 (12.5%) were positive for *S. aureus* according to the MPN method. This included 15 (12.6%) of the 119 cooked pork samples, 15 (9.8%) of the 153 cooked chicken samples, 17 (13.4%) of the 127 cooked duck samples, six (11.3%) of the 53 samples from cold vegetable dishes in sauce, 10 (19.2%) of the 52 cold noodle samples, and six (13.0%) of the 46 fried rice/sushi samples. Overall, 50.7% (35/69) of positive samples had a bacterial load of < 1 MPN/g, and 42.0% (29/69) reached 1 MPN/g. Five samples exceeded 10 MPN/g (Table 1).

**Table 1:** **Prevalence and load of *Staphylococcus aureus* in retail RTE foods in China**.

**Type of products**	**Samples tested no**.	**No. (%) Samples positive for *Staphylococcus aureus***	**No. of samples**			
			***Staphylococcus aureus*(MPN/g)**			
			**0.3–1**	**1–10**	**10–110**	**>110**
Cooked pork	119	15 (12.6)	10	5		
Cooked chicken	153	15 (9.8)	6	8	1	
Cooked duck	127	17 (13.4)	7	7	3	
Cold vegetable dishes in sauce	53	6 (11.3)	4	2		
Cold noodles	52	10 (19.2)	5	4	1	
Fired rice/sushi	46	6 (13.0)	3	3		
Total	550	69 (12.5)	35	29	5	

### Antimicrobial susceptibility of the *S. aureus* isolates

Of the 69 *S. aureus* isolates recovered, seven were confirmed as MRSA by cefoxitin disc diffusion test. Six of the isolates were *mecA*-positive, and none tested positive for *mecC* by PCR. All of the MRSA isolates were resistant to ampicillin and penicillin G, and 66.7% were resistant to clindamycin, erythromycin, tetracycline, and kanamycin. All MRSA isolates were susceptible to cephalothin, amikacin, linezolid, quinupristin/dalfopristin, and vancomycin (MICs < 1 μg/mL). The antimicrobial resistance profiles of the MRSA isolates are shown in Table 2.

**Table 2:** **Prevalence and characteristics of methicillin-resistant *Staphylococcus aureus* isolates**.

**City**	**Source**	**Sample**	**Isolate**	**Cefoxitin**	***MecA***	**Sequence type**	***SCCmec* type**	**Antimicrobial resistance profile**
Guagnzhou	Cold noodles	112	S8	+	+	ST338	V (5C2)	AMP-FOX-P-C-TE-K-E-DA
Sanya	Cold noodles	560	S27	+	+	ST3239^*^	NT (2/)	AMP-FOX-P-CN-K-SXT-E-DA-RD
Nanning	Cooked duck	678	S33	+	+	ST59	IVa (2B)	AMP-FOX-P-TE-K-E-DA
Fuzhou	Cooked chicken	2236	S51	+	+	ST9	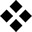 (2C2)	AMP-FOX-P-C-TE-CIP-CN-K-SXT-E-DA
Nanning	Cooked duck	2284	S53	+	+	ST59	IVa (2B)	AMP-FOX-P-TE
Nanchang	Fired rice/sushi	2512	S66	+	+	ST1	V (5C2)	AMP-FOX-P
Haikou	Cold vegetables dishes in sauce	509	S23	+	−			AMP-FOX-P-E

* *Novel sequence type. NT, not typeable. /, no amplification product. 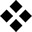, undescribed SCCmec type*.

*AMP, ampicillin; E, erythromycin; DA, clindamycin; FOX, cefoxitin; RD, rifampicin; P, penicillin G; C, chloramphenicol; TE, tetracycline; CIP, ciprofloxacin; CN, gentamicin; K, kanamycin; SXT, trimethoprim-sulfamethoxazole*.

Among the 62 methicillin-susceptible *S. aureus* (MSSA) isolates, all were resistant to at least one antimicrobial agent, and 47 isolates (75.8%) were resistant to more than three antimicrobials. The highest levels of resistance were observed for ampicillin (98.4%), penicillin G (98.4%), and tetracycline (43.5%). The antimicrobial resistance profiles of the MSSA strains are shown in Table 3.

**Table 3:** **Antimicrobial resistance susceptibility profiles of methicillin-susceptible *Staphylococcus aureus* isolates**.

**Antimicrobial agents**	**No. of isolates(%)**		
	**Resistant**	**Intermediate**	**Susceptible**
Ampicillin (AMP, 10 μg)	61 (98.4)	0 (0.0)	1 (1.6)
Cephalothin (KF, 30 μg)	0 (0.0)	0 (0.0)	62 (100)
Cefoxitin (FOX, 30 μg)	0 (0.0)	0 (0.0)	62 (100)
Penicillin G (P, 10 μg)	61 (98.4)	0 (0.0)	1 (1.6)
Choramphenicol (C, 30 μg)	4 (6.5)	1 (1.6)	57 (91.9)
Tetracycline (TE, 30 μg)	27 (43.5)	0 (0.0)	38 (56.5)
Ciprofloxacin (CIP, 5 μg)	1 (1.6)	1 (1.6)	60 (96.8)
Amikacin (AK, 30 μg)	1 (1.6)	0 (0.0)	61 (98.4)
Gentamicin (CN, 10 μg)	6 (9.7)	0 (0.0)	56 (90.3)
Kanamycin (K, 30 μg)	10 (16.1)	8 (12.9)	44 (71.0)
Trimethoprim-Sulfamethoxazole (SXT, 25 μg)	11 (17.8)	2 (3.2)	49 (79.0)
Erythromycin (E, 15 μg)	17 (27.4)	5 (8.1)	40 (64.5)
Clindamycin (DA, 2 μg)	7 (11.3)	2 (3.2)	53 (85.5)
Rifampicin (RD, 5 μg)	4 (6.5)	0 (0.0)	58 (93.5)
Linezolid (LZD, 30 μg)	1 (1.6)	0 (0.0)	61 (98.4)
Quinupristin/dalfopristin (QD, 15 μg)	0 (0.0)	0 (0.0)	62 (100)
Vancomycin (VA, MIC)	0 (0.0)	0 (0.0)	62 (100)
Pansusceptible	0 (0.0)		
≥1 Antimicrobial	62 (100)		
≥3 Antimicrobial	47 (75.8)		
≥6 Antimicrobial	9 (12.9)		

### MLST and SCCmec-typing of mecA-positive MRSA isolates

In the present study, seven phenotypically MRSA isolates were identified; however, only six isolates were *mecA*-positive by PCR. Among the six *mecA*-positive MRSA isolates, two were recovered from different cooked duck samples in the same city (Nanning). These were both identified as ST59 and harbored SCC*mec* type IVa (2B). Amongst the remaining *mecA*-positive isolates, one belonged to ST338 and harbored SCC*mec* type V (5C2), one belonged to ST1 and harbored SCC*mec* type V (5C2), and one belonged to ST9 and harbored an unreported SCC*mec* type (2C2). One novel ST (characterized as ST3239) was identified for a MRSA isolate recovered from sausage. SCC*mec* typing of this isolate was not possible as multiplex PCR-1, which provides the *ccr* gene complex type, produced a 937-bp DNA fragment, consistent with type 2, but multiplex PCR-2, which types the *mecA* gene complex class, did not amplify.

## Discussion

In recent years, the consumption of RTE foods in China has increased markedly. However, reports on *S. aureus* and MRSA contamination in these foods are scarce. The prevalence of *S. aureus* in food samples in the current study was 12.5% (69/550), which is lower than that observed (25.1%) in RTE foods (cooked meat, vegetable salads, boiled peanuts, cold noodles, and dried tofu) from Shaanxi Province, China (Wang et al., 2014). However, the prevalence of MRSA in our study (1.3%) was higher than that (0.6%) of the study by Wang et al. (2014). As our results were obtained from a large number of samples from most regions in China, the data are more comprehensive and systematic, and more representative of China as a whole.

The prevalence of *S. aureus* and MRSA in RTE foods in the current study differs from that reported in raw food products in China (Wang et al., 2014; Song et al., 2015), which generally have relatively higher levels of *S. aureus* (19.3–24.2%) and MRSA (1.7–5.6%). In addition, antimicrobial susceptibility testing revealed that not only all of the MRSA isolates, but a significant number of the MSSA isolates (47/62; 75.8%), were resistant to three or more antibiotics. The high prevalence of penicillin, ampicillin, tetracycline, and erythromycin resistance observed in our study is similar to results from previous studies in raw food products in China (Wang et al., 2014) and other countries (Rodríguez-Lázaro et al., 2015). However, the findings of our study are even more serious in terms of public health because RTE foods are consumed without further cooking, which would eliminate or reduce the microbial load. Consequently, the incidence of *S. aureus* and MRSA in RTE foods, along with the spread of antibiotic resistant strains, represents a potential health hazard to humans.

All MRSA isolates in the current study displayed resistance to three β-lactams, as well as showing high levels of resistance to clindamycin, erythromycin, tetracycline, and kanamycin. Fortunately, all of these isolates were susceptible to linezolid, quinupristin/dalfopristin, and vancomycin (MICs < 1 μg/mL), which are the few remaining effective agents for treatment of MRSA infections.

Our study provides evidence for the existence of two different lineages of MRSA in RTE foods in China: CA-MRSA and LA-MRSA. ST59 (2/6, 33.3%) was the predominant ST in our study, and its single locus variant ST338 was also detected. ST59 and ST338 belong to CC59, which is the dominant CA-MRSA CC in Asia and is a significant cause of human infection attributable to MRSA (Li et al., 2013; Chen and Huang, 2014). CC59 MRSA isolates also prevail in China, and are also the dominant clone in healthy carriers (Du et al., 2011) and in patients with community-acquired infections (Li et al., 2013). Amongst CC59 strains, two dominant SCC*mec* types (IV and V) and different antimicrobial resistance profiles have been described. A previous study showed that ST59 and ST338 were the first and second most common STs in Chinese pediatric community-acquired pneumonia, especially the dominant ST59-MRSA-IVa and ST338-MRSA-V clones (Geng et al., 2010). ST59-MRSA-IV and ST338-MRSA-V have also been associated with cases of bacteremia in China (He et al., 2013).

ST1 isolates are generally considered to be CA-MRSA (Porrero et al., 2013), although they have also been found in animals (Porrero et al., 2013). ST1 MRSA has most commonly been reported in the United States and Canada (Chuang and Huang, 2013), and has only been found sporadically in China. However, multiple ST1-MRSA-IV clones were reported to cause community-acquired pneumonia and skin/soft-tissue infections in Chinese children (Geng et al., 2010). ST1 has also been associated with staphylococcal food poisoning in Korea (Cha et al., 2006) and China (Yan et al., 2012). Some studies have shown that rates of resistance to non-β-lactam agents amongst ST1 MRSA isolates vary between countries and clones. ST1 MRSA strains in the United States usually show resistance to several non-β-lactam agents, including erythromycin and clindamycin, while MRSA strains in Australia are often uniformly susceptible to almost all non-β-lactams (Chen J. et al., 2014). The ST1 MRSA isolate in our study was also susceptible to all non-β-lactams, indicating that it might differ from that prevailing in the United States.

One ST9 LA-MRSA isolate was also identified in the current study. ST9 is the most prevalent LA-MRSA clone in most Asian countries, including China (Cui et al., 2009; Wagenaar et al., 2009; Petinaki and Spiliopoulou, 2012), despite ST398 being the most widespread ST in the rest of the world (Weese and Van Duijkeren, 2010; Petinaki and Spiliopoulou, 2012). ST9 MRSA is the predominant clone in food animals and animal-derived products (pork, chicken, and raw milk) in China (Cui et al., 2009; Wagenaar et al., 2009; Boost et al., 2013), and has also been found in farm workers (Cui et al., 2009) and associated with human infections (Liu et al., 2009; Yu et al., 2014). A previous study described ST9-MRSA-SCC*mec*V/NT isolates from patients with severe clinical illness (Yu et al., 2014). However, these patients were not livestock handlers and did not keep close contact with livestock (Yu et al., 2014), indicating that MRSA ST9 can pose a threat to humans through the food chain. ST9 MRSA strains with SCC*mec* types II (Wang et al., 2014), III (Cui et al., 2009), IVb (Boost et al., 2013; Wang et al., 2014; Yan et al., 2014), and V (Yu et al., 2014) have also been reported in China. However, the ST9 MRSA isolate recovered in our study showed considerable heterogeneity, harboring a previously undescribed SCC*mec* type with a type 2 *ccr* (A2B2) and a class C2 *mec* gene complex (2C2). These results suggest that ST9 MRSA acquired novel genomic islands during evolution from ST9 MSSA. Notably, the ST9 MRSA isolate in the current study was multidrug resistant, with resistance to β-lactams as well as eight other antibiotics widely used in chemotherapy. This finding suggests that the isolate originated from livestock.

Human infections caused by foodborne MRSA strains have been reported (Jones et al., 2002). Therefore, the potential role of food in the dissemination of successful MRSA lineages cannot be ignored. While ST59 and ST9 MRSA strains have been detected in food in China (Wang et al., 2014; Song et al., 2015), ST338 and ST1 MRSA isolates from food have never been reported. However, the previously reported ST59 MRSA strains were ST59-MRSA-II and ST59-MRSA-NT clones, not the more virulent ST59-MRSA-IVa clone identified in our study, which is associated with severe infections. Our results suggested that the presence of MRSA in RTE foods is the result of human contamination through poor personal hygiene, or through cross-contamination of carcasses during food processing. On the other hand, the MRSA clonal complexes identified in the present study are prevalent amongst clinical isolates associated with severe infection in China, suggesting food as a potential environmental source of *S. aureus* isolates with significant clinical relevance.

## Conclusion

To our knowledge, this is the first comprehensive study of the prevalence of *S. aureus* and MRSA in retail RTE foods from diverse regions of China. The present study revealed a relatively high prevalence of *S. aureus* and MRSA, and high rates of antimicrobial resistance amongst the isolates. Epidemic CA-MRSA and LA-MRSA clones associated with severe infection were identified amongst the isolates. Our data confirm the potential role of RTE foods in the dissemination of multidrug-resistant *S. aureus* strains and successful MRSA lineages in China, and highlights the health risks for consumers. Effective measures should be taken to ensure the safety of our food products.

## Author contributions

Conceived and designed the experiments: XY, QW, and JZ. Performed the experiments: XY and SY. Analyzed the data: XY and JZ. Contributed reagents/materials/analysis tools: XY, JZ, WG, JH, and SC.

## Funding

This work was supported by the Science and Technology Projects of Guangdong (2014B050504007, 2013B020312001).

### Conflict of interest statement

The authors declare that the research was conducted in the absence of any commercial or financial relationships that could be construed as a potential conflict of interest.
